# Response to the critical analysis of the article ‘Comparison and convergent validity of five Mediterranean dietary indexes applied to Brazilian adults and older adults: data from a population-based study (2015 ISA-Nutrition)’

**DOI:** 10.1017/jns.2023.60

**Published:** 2023-07-17

**Authors:** Amália Almeida Bastos, Paula Victória Félix, Michelle Alessandra de Castro, Regina Mara Fisberg, Mary Yannakoulia, Sandra Maria Lima Ribeiro

**Affiliations:** 1 Department of Nutrition, Public Health School, University of Sao Paulo, São Paulo, Brazil; 2 School of Feeding Coordination, São Paulo City Hall, São Paulo, Brazil; 3 Department of Nutrition and Dietetics, School of Health Sciences and Education, Harokopio University, Athens, Greece; 4 School of Arts, Sciences and Humanities, University of Sao Paulo, São Paulo, Brazil

We appreciate the careful reading and critical aspects brought by Cheo *et al.* (in press)^(1)^ of our article titled ‘*Comparison and convergent validity of five Mediterranean dietary indexes applied to Brazilian adults and older adults: data from a population-based study (2015 ISA-Nutrition)*’^(2)^. As a subject still poorly explored in Brazilian populations and as connoisseurs of the characteristics of the database, bringing our point of view regarding their additional comments is pertinent.

Different population characteristics act as dietary determinants. However, most research with this approach, including those cited by the authors, directed at the Mediterranean dietary pattern (MDP), involves Mediterranean populations^(3–5)^. Even referring to cross-sectional data focused on the Brazilian population, it is a robust study working with a representative sample of the foremost industrial centre in Brazil and the largest population in the Americas. In addition to probably differing when considering a Brazilian sample, not only such factors influence adherence to the MDP, but also the diet quality based on the Brazilian Healthy Eating Index-Revised (BHEI-R) as shown by Aline *et al.*^(6)^ that investigated the same sample as our study. Thus, this critical comment would also apply to studies with the same objective involving other dietary patterns. Also, as in most studies, the investigation is around a sample of a specific country. However, as our sample is restricted to the Brazilian city of São Paulo, recognised as a limitation of our study, we carefully reinforced the impossibility of generalising our results to other countries and other regions of Brazil.

The study cited by the authors, which points to a high prevalence of substance abuse in Brazil, apparently included a younger population (aged 12–65 years old) compared to our study (aged 20–95 years old). The consumption of illicit drugs in 2015 in Brazil was estimated at 3⋅2 %, and dependents are 0⋅8 % of the population. In addition, the same data show a higher prevalence (7.4 %) at younger ages (18–24 years old) and less prevalent (1.1 %) at older age groups (55–65 years old)^(7)^. We consider that these data are irrelevant to the point of compromising our results.

Regarding eating disorders, the Global Burden of Diseases^(8)^ reported a prevalence of only 0⋅25 % in Brazilians, dropping to 0⋅16 % when considering only individuals over 20. In the sample we investigated from the 2015 Health Survey of São Paulo with Focus on Nutrition Study (2015 ISA-Nutrition)^(9)^, some variables linked to the behaviour of people with eating disorders were collected; we now bring their description. Regarding body weight dissatisfaction, 62 % of the sample referred to not adopting any strategy to lose weight. Still, 95 % reported not changing their eating habits or following a modified diet. We do not consider this aspect an essential exclusion factor based on such facts. The same can be thought about mental health status, as 85 % of the sample is unaffected by any mental disorder.

Socioeconomic status and food environment influence diet, and some studies within this perspective, have already been carried out with data from the 2015 ISA-Nutrition. The *per capita* household income was identified as the main determinant of inequality in diet quality in adolescents, adults and older adults^(6)^. Among adolescents, higher family income (OR 2⋅56; CI 95 % 1⋅47, 4⋅45) and street markets closer to households (OR 1⋅73; CI 95 % 1⋅01, 3⋅00) were associated with higher consumption of fruits and vegetables^(10)^. We are unaware of studies carried out in the context of the Mediterranean diet in Brazilian samples.

Admittedly, the pandemic has resulted in changes in lifestyle across the world. This limitation is part of all investigations carried out before this period. After this period, studies with the Brazilian population showed changes varying between greater consumption of fast food and instant meals^(11)^, using more food delivery services, and prioritising homemade meals and fresh food^(12)^. This is an unquestionable factor that needs to be further clarified by future studies.

Finally, it is important to mention that despite the influence of such factors on adherence to the MDP, all the mentioned factors equally affected each Mediterranean dietary index since the same sample and methodology were applied to every Mediterranean dietary index. Assessing barriers to food access and other factors affecting adherence to the Mediterranean diet goes beyond the aim of the present analysis. However, we appreciate the comments, as a starting point for a future analysis. Thus, the points addressed by the authors brought pertinent analyses on the topic of our research.
